# A community-informed approach to develop a gardening model for the Bangladeshi community in Brooklyn, NY

**DOI:** 10.1093/heapro/daag065

**Published:** 2026-05-25

**Authors:** Sze Wan Chan, Fatema Kamal, Rhyden Dowd, Sabiha Sultana, Nadia S Islam, Stella S Yi

**Affiliations:** Section for Health Equity, Department of Population Health, NYU Langone Health, 180 Madison Ave, New York, NY 10016, United States; Section for Health Equity, Department of Population Health, NYU Langone Health, 180 Madison Ave, New York, NY 10016, United States; Brooklyn Grange, 63 Flushing Ave, Building 3, Suite 1105, Brooklyn, NY 11205, United States; Section for Health Equity, Department of Population Health, NYU Langone Health, 180 Madison Ave, New York, NY 10016, United States; Section for Health Equity, Department of Population Health, NYU Langone Health, 180 Madison Ave, New York, NY 10016, United States; Section for Health Equity, Department of Population Health, NYU Langone Health, 180 Madison Ave, New York, NY 10016, United States

**Keywords:** Bangladeshi, Bangladeshi American, gardening, vegetables

## Abstract

Nationally, there is increased investment in interventions that address diet-related chronic diseases however few studies and interventions are developed to reflect the values and lifestyles of many communities, presenting a barrier to participation. This study aims to better understand the motivators and barriers for the Bangladeshi American community in Brooklyn, New York to participate in diet-related interventions. Formative qualitative interviews were conducted in English and Bangla with Bangladeshi adults (*n* = 12) to understand current shopping/cooking behaviors, access and usage of food programs, awareness and interest in food programs, and future program preferences. Participants reported three key themes: centrality of community behaviors for program acceptability, creating opportunities to leverage community and social motivations, and addressing logistical concerns during program development. Gardening emerged as a promising program offering to increase access to fresh produce, strengthen community bonds, and foster cross-cultural understanding. Using community feedback, Harvest Share Seedlings, a community-informed home gardening program, was co-developed and implemented with farming and community partners to increase access to fresh produce for the Bangladeshi community. The findings highlight the need to understand and center community-specific considerations when designing and implementing food programs and interventions. Adopting a community-informed approach increases uptake and acceptability from the community, and ensures sustainability in the long run.

Contribution to Health Promotion

- Demonstrates the value of using a community-informed approach that engages local stakeholders for health promotion program development.
- Highlights the importance to center culture and preferences of local communities to ensure uptake and acceptability of food programs.
- Identifies key barriers to food program participation in the Bangladeshi community, including lack of language support, transportation and distance, and affordability of produce.

## Background

Health disparities in chronic diseases are prevalent across various ethnic and socioeconomic groups, exacerbated in part by structural and social inequities such as access to food and essential services (Cockerham *et al*. 2017, LeCroy *et al*. 2023, Town *et al*. 2024). Nationally in the United States (USA) there is increased investment in interventions that address diet-related chronic diseases, which commonly includes medically tailored meals, medically tailored food packages, produce prescription programs, nutritious food referrals, but can also include meal delivery programs, community supported agriculture (CSA), fruit and vegetables incentives, and community gardens (Center for Health Law and Policy Innovation of Harvard Law School 2020, Bleich *et al*. 2023). Early research shows the promise of food-related interventions in improving fruit and vegetable consumption, and reducing food insecurity (Downer *et al*. 2020, Little *et al*. 2021, LeCroy *et al*. 2023). However, few studies and interventions are tailored to immigrant communities’ culture and lifestyles, presenting a barrier to participation in such programs. Further, not all programs prioritize to uplift local food producers and farms (Yi, Lee, *et al*. 2021; McWhorter *et al*. 2022). Given the growth of immigrant populations in the USA in recent years and the high burden of diet-related disparities they experience (Commodore-Mensah *et al*. 2018; Gramlich and Passel 2024), it is especially important for food programs to use community-engaged methods, incorporate culturally appropriate food access and nutrition information, and meaningfully engage with both community and farm-based partners.

The PEN-3 model is originally created for the planning and development of culturally appropriate health education programs. It has been widely used to understand the relationship between culture and health across different topics including HIV, cancer, hypertension, diabetes, malaria, nutrition, and smoking (Airhihenbuwa 1990, Iwelunmor *et al*. 2014). The PEN-3 model consists of three dimensions of health beliefs and behaviors that are interrelated and interdependent. The health education dimension consists of deciding the focus of the program and whether it is person, extended family, or neighborhood. The educational diagnosis dimension consists of examining whether health behaviors are predisposing, enabling, or nurturing. Finally, the cultural appropriateness dimension categorizes health beliefs and behaviors are positive, exotic, or negative (Airhihenbuwa 1990). As a model, it offers a guiding framework to align food-related interventions with cultural preferences of different immigrant populations by integrating culturally relevant factors in the development of interventions (Iwelunmor *et al*. 2014).

The Bangladeshi population is one of the three largest South Asian groups in the USA. Among South Asians, Bangladeshi immigrants constitute one of the newest and fastest-growing immigrant communities, with nearly half of all Bangladeshi immigrants in the USA residing in the New York metropolitan area (Patel *et al*. 2012, Akhter and Yang 2023). The majority of the Bangladeshi community in New York City (NYC) are also born outside the USA, prefer to communicate in Bangla vs. English, live in poverty, and live in overcrowded households; 34% of Bangladeshi individuals in NYC are facing food insecurity according to locally collected data (Asian American Federation 2020, Islam *et al*. 2022, Nguyễn *et al*. 2024). Disaggregated data on the Bangladeshi subgroup is limited in the health literature, but data on South Asian American communities in aggregate indicate a high prevalence of cardiovascular disease, diabetes, hypertension, and overweight/obesity (Koirala *et al*. 2021, Kandula *et al*. 2023, Rich *et al*. 2023). Culturally, gardening is a common practice for Bangladeshi populations. In Bangladesh, home gardening has historically been promoted for decades by international and local non-governmental organizations as well as the Bangladeshi Agricultural Research Institute to increase consumption of healthy foods within households (Schreinemachers *et al*. 2015, Baliki *et al*. 2019). Taking the health behavior dimension of the PEN-3 model into consideration, gardening presents as a culturally aligned diet-related intervention for the Bangladeshi community.

Within the literature, community gardens and gardening-related interventions have been reported to offer a wide range of observed benefits including physical, nutritional, mental, social, and economic. In the physical and nutritional domains, gardening was described to increase physical activity and time spent outdoors and decrease sedentary behavior (Palar *et al*. 2019, Beavers *et al*. 2022, Kersten *et al*. 2023, McCartney *et al*. 2023). Additionally, gardening was reported to increase consumption of fruits and vegetables, increase access to healthier foods, improve sleep and chronic disease conditions (e.g. hypertension and blood sugar), and lower body mass index and weight (Palar *et al*. 2019, Shin *et al*. 2020, Beavers *et al*. 2022, Hume *et al*. 2022, Kersten *et al*. 2023, McCartney *et al*. 2023). For mental health impacts, gardening was reported to reduce stress and provide participants with a sense of joy or relaxation (Palar *et al*. 2019, Beavers *et al*. 2022, McCartney *et al*. 2023). Social benefits included fostering social connections, greater family involvement and interactions around food, and sharing knowledge and produce with neighbors (Beavers *et al*. 2022, Kersten *et al*. 2023). For immigrant populations in particular, gardens were reported as sources to access traditional foods with high nutritional value and cultural significance (Onyango *et al*. 2025). Lastly for economic impacts, gardening was found to improve food security and reduce grocery bills, especially during harvest seasons (Palar *et al*. 2019, McCartney *et al*. 2023).

Despite the growing Bangladeshi population in NYC and their unique cultural profile, there is a lack of comprehensive reviews and studies focusing on their diet-related needs and challenges, nor any formative assessment of the fit of food programs including community gardens or gardening-related interventions for this group (Patel *et al*. 2012). The aim of this study is to design a culturally appropriate food program for the Bangladeshi American community in Brooklyn. The driving question behind this study asks “what are the motivators and barriers for the Bangladeshi American community in Brooklyn to participate in diet-related interventions.”

## Methods

### Context and rationale for this study

Harvest Share is a partnership between NYU Langone Health and 10 multi-sector partners. The goal of Harvest Share is to implement a whole-of-community intervention in Sunset Park and surrounding neighborhoods in Brooklyn to improve diet and social and built environments for English-, Chinese-, Spanish-, and Bangla-speaking communities. The cornerstone of Harvest Share is a culturally tailored CSA produce box featuring Chinese and Mexican vegetables, which launched in 2022 (Chan *et al*. 2024).

In 2023, we sought to recruit Bangladeshi community members living in Kensington to the Harvest Share CSA. Kensington is a neighborhood next to Sunset Park with a significant Bangladeshi population and is roughly 3 miles from the Harvest Share CSA pick up site in Sunset Park, which is approximately a 16 minute drive by car or 45 minute public transportation commute (Asian American Federation 2015, Google Maps 2026). This expansion was based in part from the NYC COVID-19 Community Health Resources and Needs Assessment, which found that 77% of Bangladeshi individuals needed help getting food during the pandemic (Ðoàn *et al*. 2022). Knowledge and guidance from NYU’s community health workers with deep knowledge of Bangladeshi culture and local food ways also informed the focus on the addition of Bangladeshi community members to the Harvest Share CSA. There is often overlap and co-location of Asian ethnic food markets in NYC neighborhoods so community members may be shopping across different types of markets that contain common produce across ethnicities (i.e. Chinese and Bangladeshi), thus contributing to the Bangladeshi community’s familiarity with and usage of Chinese produce (e.g. bok choy and Chinese bitter melon). Although the Harvest Share CSA featuring Chinese vegetables is not specifically designed for the Bangladeshi community, the research team decided to trial acceptability of the CSA without any modifications for Bangladeshi families, especially for the potential resource and cost-saving implications.

In the spring of 2023, Bangladeshi community health workers actively shared flyers translated into Bangla to their networks and a bilingual intern emailed or called participants from the DREAM Initiative willing to be recontacted to discuss the Harvest Share CSA (Lim *et al*. 2021). The outreach team reported low interest from the community and no Bangladeshi community member signed up for the Harvest Share CSA. Transportation barriers and lack of time were some of the reasons given to the research team for individual’s non-participation. We therefore conducted formative research to understand the motivators and barriers for the Bangladeshi community in Brooklyn to participate in a food program.

### Approach

We used a qualitative study design underpinned by community-engaged research (CEnR) principles and an assets-based approach to health promotion. CEnR is a process where researchers collaborate with communities and directly incorporate their voices and priorities into the research process, and an assets-based approach intentionally engages the community and other relevant stakeholders to identify and use existing community strengths to improve health (Centers for Disease Control and Prevention, n.d.; Blickem *et al*. 2018, Martin-Kerry *et al*. 2023). This approach was chosen to allow the study team to understand the exact motivators and barriers of the Bangladeshi community in order to increase accessibility of the modified Harvest Share program. This approach was also selected as it enabled incorporation of existing resources and networks available to Harvest Share partners during program design.

### Participants and recruitment

A purposive sample of Bangladeshi individuals were recruited by leveraging rich existing relationships that both Brooklyn Grange and NYU Langone Health had with community and faith-based organizations and a local community garden. Brooklyn Grange is a farm partner growing Chinese vegetables for the Harvest Share CSA. Brooklyn Grange has built trust among Bangladeshi community gardeners over the course of several seasons, propagating family heirloom varieties in their greenhouse and then distributing those seedlings back to the gardeners to grow in family plots. This familiarity helped participants feel more trusting and open to provide feedback for the interviews. Participants were eligible if they: (i) were 18 years or older, (ii) self-identified as Bangladeshi/Bangladeshi American, (iii) lived in Brooklyn, New York, (iv) were able to speak English and/or Bangla, and (v) were interested in participating in a food program. English and Bangla flyers with information about the study and eligibility criteria were distributed by the organizations to community members. Interested individuals then contacted the study team in their preferred language (English or Bangla) to confirm their eligibility. Community members were also approached by bilingual staff at partner community organizations through routine and scheduled programming and other events to share the study flyer.

### Data collection

Verbal informed consent and permission to audio-record was obtained over the phone prior to each interview. Interviews lasted 30–60 minutes and were conducted in English or Bangla over Zoom (either phone or computer) based on participant preference by trained bilingual staff. All interviewees chose to complete the interview over the phone.

Interview guide development was loosely informed by the PEN-3 model—questions and prompts were chosen to allow the research team to better understand participants’ beliefs and behaviors related to diet and food access at the person, extended family, and neighborhood level, and also to identify predisposing, enabling, and nurturing factors. Interview questions aimed to open up discussion in relation to participants’: (i) current shopping and cooking behaviors, (ii) access and usage of food assistance programs, (iii) awareness and interest in food programs (community gardens, CSAs, and farmers’ markets), (iv) program preferences (location, payment options, and produce offerings), and (v) interest in future leadership opportunities. Participants received a $40 gift card as a token of appreciation for their time and for completing the interviews. All study procedures were approved by the Institutional Review Board at NYU Langone Health.

### Study team

Our research team acknowledges the potential individual subjectivity and positionality in the research. The semi-structured interview guide was co-developed by S.W.C. and F.K. with Brooklyn Grange (Supplemental File S1). Three members of the team (F.K., S.S., and J.U.) conducted interviews. Interview protocol training was provided by S.W.C., a project coordinator with experience in qualitative research and diet/nutrition intervention implementation, who identified as a Chinese female. F.K., S.S., and J.U. all identified as Bangladeshi American, were bilingual in English and Bangla, and had experience recruiting and engaging Bangladeshi community members in research projects focused on diet-related health promotion. The research team may have had some prior assumptions of the Bangladeshi community’s preferences and barriers to participate in a food-related intervention and an awareness of what is feasible in future program design. The interview guide was thus purposely kept broad in order to capture beliefs and behaviors of interviewees beyond the team’s assumptions.

### Data analysis

Interviews conducted in English were transcribed verbatim. Interviews conducted in Bangla were first transcribed in Bangla, and then translated into English by bilingual staff (F.K., S.S., and J.U.). Transcriptions and translations were double-checked against original audio-recordings for accuracy and completeness.

Data analysis utilized an adapted approach to Braun and Clarke’s thematic analysis (Braun and Clarke 2006). Because our research question had a specific aim to understand community motivators and barriers to participate in diet-related interventions, the focus of analysis was guided by an assets-based lens to identify existing habits, networks, and cultural practices within the community. S.W.C. was the lead coder and read all the transcripts in their entirety before developing a preliminary codebook with codes focused on community experiences and specific program preferences. S.W.C. then met with the rest of the coding team (F.K. and J.C.) to discuss the preliminary codebook and refine wording of codes, sub-codes, and definitions as necessary. All transcripts were then double blind coded (S.W.C., F.K., and J.C.) using Dedoose qualitative data analysis software (Version 9.0.107, Los Angeles, CA: SocioCultural Research Consultants, LLC) (“Dedoose,” n.d.). For each transcript, two coders met after individually coding to discuss their own interpretations of the data and resolve discrepancies in coding. The coding team also met weekly to iteratively update the codebook (Supplemental File S2) by discussing refinement of existing codes and as necessary, any new codes that should be added. For example, initial coding by the team resulted in sub-codes “interest in community gardens” and “interest in CSAs” being divided into sub-sub-codes such as “social” or “health” to track specific motivators of the community. Once all transcripts were coded, the coding team met to discuss key themes. Each coder then individually sorted relevant coded data extracts by each theme. The coding met a final time to discuss the sorted data extracts and refine final themes guided by the lived experiences of Bangladeshi study team members.

## Results

A total of 12 participants were interviewed. Participant and household characteristics are summarized in Table 1. The average age was 43 years (range: 21 to 60 years) and the average years lived in the USA was 18 years (range: 3 months to 32 years). Participants were equally female (50%) and male (50%); no participants chose non-binary. Most spoke Bangla at home (67%) and had three adults (36%) and two children (55%) living in the household. Participants were most interested in farmers’ markets (92%) and community gardens (83%) for programming. Participant motivations and barriers to participating in a food and gardening program specific to the Bangladeshi American community are presented via three key themes: (i) centrality of culture for program acceptability, (ii) creating opportunities to leverage community and social motivations, and (iii) addressing accessibility concerns during program development.

**Table 1:** Individual and household characteristics of Bangladeshi-identifying participants (*n* = 12).

Characteristic (*n*)	Mean (range)
Age (11)	43 (21–60)
Years lived in the USA (10)	18 (0.25–32)

Characteristic (*n*)	*n* (%)
Self-identified gender (12)	
Male	6 (50)
Female	6 (50)
Non-binary	0 (0)
Languages spoken at home (12)	
Bangla	8 (67)
English and Bangla	4 (33)
Number of adults living in household (11)	
1	2 (19)
2	1 (9)
3	4 (36)
4	3 (27)
5	1 (9)
Number of children living in household (11)	
0	4 (36)
1	1 (9)
2	6 (55)
Usage of food assistance programs (12)	
Yes	7 (58)
No	5 (42)
Familiarity with SNAP^a^ (12)	
Yes	12 (100)
No	0 (0)
Familiarity with WIC^b^ (12)	
Yes	5 (42)
No	7 (58)
Interest in food program^c^ (12)	
Farmers’ market	11 (92)
Community garden	10 (83)
CSA	8 (67)

^a^SNAP = Supplemental Nutrition Assistance Program.

^b^WIC = Special Supplemental Nutrition Program for Women, Infants, and Children.

^c^Interviewees were permitted to select multiple response options so percentages do not total 100%.

### Centrality of culture for program acceptability

All participants heavily emphasized the importance of cultural alignment and appropriateness to increase the acceptability and appeal of a food and gardening program designed for the Bangladeshi community. Participants’ cooking behaviors and dietary preferences are deeply rooted in Bangladeshi culture, with a strong preference for traditional Bangladeshi vegetables. When asked what vegetables they would like to receive or grow, participants listed many culturally specific vegetables such as bottle gourd, bitter melon, ridge gourd. Although participants also listed more commonly consumed vegetables across cultures such as squash, cabbage, and eggplants, these vegetables were widely used in Bangladeshi cuisine. The full list of preferred vegetables is listed in Table 2.

**Table 2:** Vegetable preferences of Bangladeshi-identifying participants (*n* = *12*).

Culturally specific vegetables	Commonly available vegetables
Bottle gourd	Cabbage
Sweet pumpkin (Mishti Lau/Kumra)	Potatoes
Flat beans (Seem)	Cucumbers
Snow peas	Squash
Asparagus beans (Borboti)	Green Spinach
Ridge gourd (Jinga)	Radish
Snake gourd (chichinga)	Green beans
Long gourd (Lau)	Carrot
Steam amaranth leaf	Broccoli
Malabar spinach (Pui shaak)	Cauliflower
Pumpkin gourd leaf (Lau shaak)	Eggplants
Red spinach (Laal shaak)	Lima beans
Okra (Darosh)	
Bitter melon (Korolla)	

Additionally, the importance of culturally appropriate foods was also reflected in participants’ grocery shopping behaviors. Participants explained that provision of halal meat options and a diverse selection of Bangladeshi vegetables were the main reasons influencing where they went grocery shopping. One participant explained that they would travel over an hour to Bangladeshi grocery stores to have “*access to the Bangladeshi fish and vegetables that we frequently eat*.” Another participant also noted how even when they find culturally appropriate produce, “*the breed and variety that is available here is vastly different from the potol [pointed gourd] breed found in Bangladesh. I really miss Bangladeshi potol*.”

Another cultural aspect found to be important to the Bangladeshi community in the design of a food program is incorporation of gardening. Eight participants (66.7%) reported having gardening experience both in Bangladesh and the USA. Many participants acknowledged the presence of community members who cultivate gardens in their balconies, backyards, and shared spaces. The majority (*n* = 10) expressed interest in engaging with a community garden to continue gardening practices. Across several interviews, participants discussed the sentimental value of gardening including the joy of growing one’s own vegetables and the passing down of family traditions.

*Yeah, there are some family values. I would say [gardening practices] were passed down because when I was growing up, [parents] would let me help them and, you know teach me a lot of things [about gardening]. So I built upon that. (33-year-old male)*

*I know a lot of people who own houses in NYC, and just garden and cultivate some vegetables during the summertime season. So in their backyard or front yard, they just cultivate some vegetables, like seasonal vegetables. It seems that if people own a house, they tend to cultivate vegetables in whatever space they have available, particularly during the summer months. (60-year-old male)*

In addition to the sentimental motivations mentioned by participants, they were also motivated to garden and grow their own food due to their preference for fresh and organic vegetables. Although participants had access to culturally appropriate vegetables in Bangladeshi grocery stores, participants noted that they were often imported and frozen. Participants gardening experience and motivations revealed that in addition to the importance of culturally appropriate vegetables, vegetable quality is also central to increase program acceptability.

*You can get access to organic vegetables through gardening on your own accord. Usually organic produce is very expensive at the grocery stores. There are also a lot of health benefits with eating organic produce you grow yourself as opposed to what we get at the grocery store. These reasons are why I am so interested in gardening. (51-year-old male)*

*The feeling of growing and harvesting your own vegetables is unmatchable with any other feeling. I get access to fresh vegetables that are otherwise so expensive to get at the grocery store. I can prepare and have them all year round, too. (42-year-old female)*

### Creating opportunities to leverage community and social motivations

Community and social factors were significant motivators for the Bangladeshi community to participate in food programs. Participants saw community gardens not long as a space to access culturally appropriate vegetables, but also somewhere they could leisurely spend time with their family and neighbors. For these participants, community gardens were seen as a way to foster community bonds and support one another.

*I want to meet people. I think there is one community garden here [Kensington], but it’s very small. I would want to meet people and want to do more community gardening. (33-year-old male)*

In addition to social motivators, participants expressed a strong desire to contribute to the community by growing and sharing vegetables. A participant who currently gardens shared that “*all the things that I produce I know are not just for my personal use. We do give out as much as possible to my network and family members.*”

The spirit of giving back to the community is reflected in participants’ various ideas of how to use produce grown in a community garden such as distributing produce through mosques and soup kitchens, and sharing with family, neighbors, and the broader community.

*If we could do something with the vegetables we harvest, that would be good, too. Maybe giving vegetables to soup kitchens? My old neighborhood had a garden at the masjid, but it was to raise funds for the masjid. (42-year-old female)*

*I wouldn’t want to sell [produce from community garden]. I would rather give it to the community members. (51-year-old male)*

Participants also emphasized the importance of creating food programming that provides spaces for community interaction, reinforcing the close-knit nature of the Bangladeshi community and highlighting opportunities to foster cross-racial and cross-ethnic connections.

*Our Bangladesh community can benefit from [a community garden], and even the other ethnicities living here, too… I think having a space where we can garden but also spend some leisure time with our families will be good. (51-year-old male)*

### Addressing accessibility concerns during program development

Participants discussed several barriers that affected accessibility to program participation. Specifically, language, location, and cost were top issues that participants viewed as factors that influenced whether they or their family/friends participated in food programs. Lack of language support was cited as a salient barrier impacting accessibility and knowledge of food assistance programs (e.g. WIC) and willingness to participate in food programs. Participants noted that limited English proficiency among immigrant communities such as the Bangladeshi community meant that individuals were often exposed to uncomfortable and unfamiliar situations to join not just food programs, but also to access health care. Two-thirds of participants spoke only Bangla at home, and they emphasized the necessity to have language support, including translated materials, interpretation services, and bilingual staff for them to feel confident participating in and successful join a food program.

*[Food assistance programs] need to have culturally relevant fruits and foods, and also if they have language*  *…*  *for me, personally, like I said, I have good English skills, but a lot of my other family members, they cannot speak English. So they need at least somebody to understand Bengali*  *…*  *I think a lot of people also never are not aware of a lot of these things [availability of food assistance programs]. Some of them are, but a lot of them are not because they just can't speak English. (33-year-old male)*

**Figure 1:**
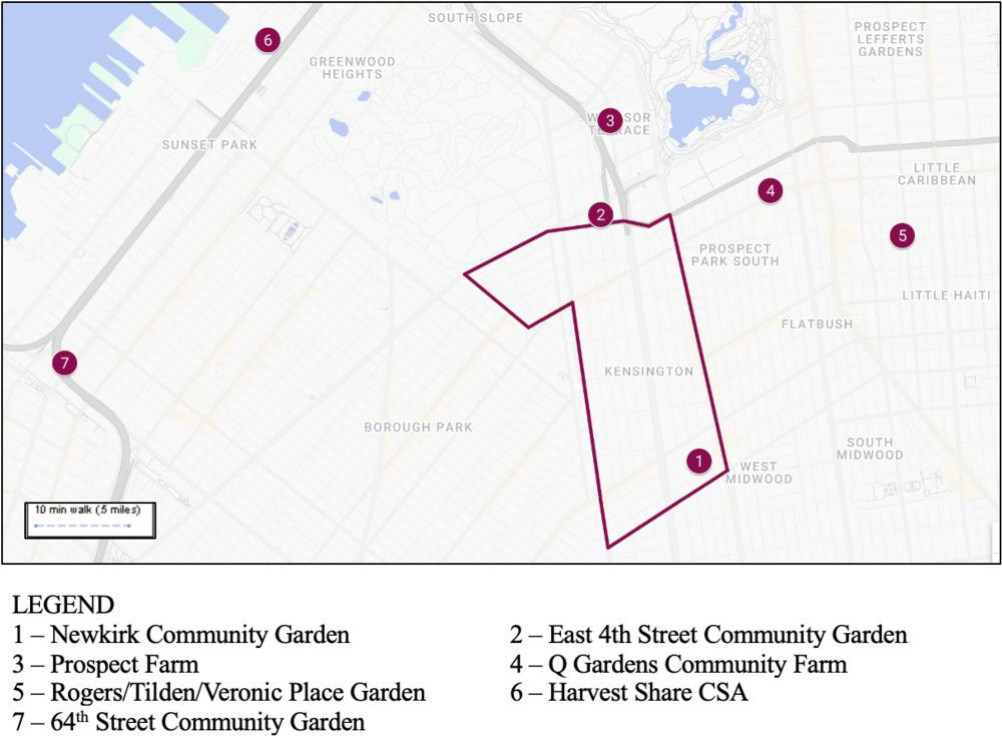
Locations of nearby community gardens and Harvest Share CSA to Kensington.

Location was another barrier affecting accessibility of food programs for the Bangladeshi community. Participants specified that food and gardening programs should be located 10 to 20 minutes walking distance within the neighborhood participants resided in, which is essential for reducing travel time and associated costs. There is currently only one community garden located within the boundaries of the Kensington neighborhood (Fig. 1), meaning that participants have to travel beyond what they indicated they are comfortable with to join a food and gardening program.

*If [the community garden] is near to me, then perhaps I would be interested. It is difficult to travel far to take care of a garden and if you are not able to yield enough produce, either. (51-year-old male)*

*If [a food program] is located near my housing, I can join but if it is far from my location, it will be difficult for me to go there. (56-year-old female)*

Affordability is another significant concern, especially given inflation—the effects of which Bangladeshi participants felt both during and after the COVID-19 pandemic. Many participants mentioned the rising cost of groceries and felt that the prices they were paying for culturally produce at grocery stores did not accurately reflect their perceived value or quality. Cost concerns were still salient even for participants that received SNAP benefits, highlighting a food affordability crisis to access culturally appropriate foods. This barrier emphasized the need to design a food program that either allowed them to purchase culturally appropriate food at lower prices or to have space and receive culturally appropriate seedlings to grow their own vegetables.

*The amount [my mother] receives is not enough. Mother receives about $280 a month, and it is not enough for the Deshi foods she enjoys eating. Everything is so expensive at the Deshi grocery. The amount she receives is not sustainable for Deshi foods. (21-year-old male)*

*As I mentioned, food prices are only going up. At Bangladeshi grocery stores, I find myself spending so much on so little and I am left with very little money for the rest of the month. [My SNAP benefits] is not very sufficient for a month. (32-year-old female)*

*When red spinach and palang shak [green spinach] comes into season, grocery stores sell it at $8–9/lb. This is outrageous. I realized that I can easily grow these vegetables on my own. (51-year-old male)*

## Discussion

Our formative research revealed the motivators and barriers for the Bangladeshi community in Brooklyn to participate in diet-related programs using qualitative interviews. Findings indicate the centrality of culture to increase program accessibility. Participants strongly indicated a preference to receive traditional vegetables such as bottle gourd, bitter melon, or ridge gourd. This predisposing nutrition behavior is consistent with prior literature on importance of provision of culturally appropriate foods when implementing food programs geared toward racial/ethnic minorities (Airhihenbuwa 1990, Taylor and Lovell 2015, Kuehn 2019, Diekmann *et al*. 2020, Lu *et al*. 2023, Chan *et al*. 2024). However, enabling factors such as the inaccessibility of culturally specific vegetables in many parts of NYC results in community members traveling long distances to reach stores that carry cultural items, highlighting a structural inequity that must be addressed. Noting the phenomenon of traveling farther to shop exclusively at ethnic grocery stores has also been observed in the Chinese American community in NYC, indicating the broader generalizability of this to immigrant communities seeking specific types of cultural foods or for language support at the store (Airhihenbuwa 1990; Yi, Russo, *et al*. 2021). To bridge this gap, the study team decided to prioritize inclusion of culturally appropriate foods in program design to enhance dietary satisfaction and foster greater participation and retention.

Our findings also reveal additional cultural aspects that need to be considered during program development. Gardening was cited as a common practice within the Brooklyn Bangladeshi community, similar to practices from their home countries (Schreinemachers *et al*. 2015, Baliki *et al*. 2019). Gardening was also cited as a way to satisfy participants’ preference for fresh and organic produce. Previous literature has found community gardens to improve access to healthy fresh foods and access to culturally relevant foods (Draper and Freedman 2010, Gray *et al*. 2022, Onyango *et al*. 2025). These findings point toward a need to integrate gardening components in our program design.

Community and social aspects emerged as powerful motivators for the Bangladeshi community, and were seen as leverage points to design a program that extends beyond the individual level and create opportunities to also focus on extended family and the neighborhood (Airhihenbuwa 1990). Participants discussed a desire to contribute back to their community by distributing harvested produce with mosques, soup kitchens, and community events. Within the literature, community gardens have been previously reported to foster psychosocial and community outcomes (Draper and Freedman 2010, Lampert *et al*. 2021, Hume *et al*. 2022, New York State Department of Agriculture and Markets 2023). Participants also viewed community gardens as not only beneficial for the Bangladeshi community, but for their neighbors of other race/ethnicities at large. Emerging research points toward the cross-racial element of community gardens and urban farms (Shinew *et al*. 2004, Mejia *et al*. 2020, New York State Department of Agriculture and Markets 2023, Zail 2023). The nature of social interactions and cooperation inherent within community gardens aligns well with interpersonal- and community-level motivations of the Bangladeshi community to participate in food programs. These motivations also reflect an asset-based approach toward health and well-being that can be the building blocks for a sustainable program driven by the community’s own priorities and strengths (Blickem *et al*. 2018, Martin-Kerry *et al*. 2023).

Finally, accessibility of programs in terms of language support, proximity, and affordability were key concerns for the Bangladeshi community. In NYC, more than half of the Bangladeshi population prefer Bangla over English in certain settings or are unable to speak English (Asian American Federation 2020). Language barriers were found to impede the Bangladeshi community’s ability both to access food assistance programs but also participation in a food-related intervention. This is in line with existing research finding lack of language support as a major barrier for immigrant communities to access health care and food (Becerra *et al*. 2018, Pandey *et al*. 2021, Yi, Russo, *et al*. 2021, Lu *et al*. 2023). In terms of proximity, most participants indicated that participation in any type of programming was contingent on proximity to their immediate neighborhood. Other enabling factors such as affordability of produce, especially due to inflation and the economic impact of the COVID-19 pandemic, was another concern (New York State Office of Budget Policy and Analysis 2024). Given these barriers, the study team centered location of programming and costs in relation to retail environments at the design stage to address the needs of the Bangladeshi community and address barriers to participation. The study team also adopted a language justice approach, including bilingual staff and transcreated program materials to reduce barriers and achieve true engagement (Nápoles and Stewart 2018).

Taken altogether, findings from key informant interviews will inform modifications to the Harvest Share program specific to the Bangladeshi community. Given the motivators and barriers of the Brooklyn Bangladeshi community, Harvest Share designed a gardening-based program, Harves Share Seedlings, located directly in Kensington that centers around growing culturally appropriate crops to increase access to fresh produce and address determinants of food insecurity. Due to the complexity of finding space and obtaining necessary permissions to start a community garden in NYC, the Harvest Share team opted to provide seedlings to Bangladeshi community members for home gardening. Brooklyn Grange propagated culturally appropriate seedlings in their greenhouse that will be distributed at a one-time event at the start of the growing season. Table 3 lists the seedlings that were grown by Brooklyn Grange. The study team worked alongside new partners based in Kensington—Public School 230 (P.S. 230) and The Singing Winds (a community organization focused on activating public spaces)—to spread the word among Bangladeshi community members about the seedling distribution event. Distribution events were staffed by a bilingual intern from NYU Langone Health, a Brooklyn Grange farmer, and the founder of The Singing Winds, which ensured that each event had language support and gardening expertise. The location of the distribution events was held at Albemarle Playground, located adjacent to P.S. 230 and a centralized location for Bangladeshi community members to gather in Kensington. In June 2024, Harvest Share successfully held its first distribution event, handing out 3172 seedlings at no cost to Kensington community members. In May 2025, the team replicated the program and a second distribution event was held with 2772 seedlings distributed at no cost. Harvest Share will host a third distribution in May 2026 and the study team will plan a qualitative evaluation to assess program experience, home gardening, behavioral changes, social outcomes, and cost-saving implications.

**Table 3:** Seedlings distributed to Bangladeshi community 2024–5.

Culturally specific vegetables	Commonly available vegetables
Holy basil	Tomato
Okra	Eggplant
Hyacinth bean	Sweet pepper
Asparagus bean	Chili pepper
Long bean	Cucumber
Winged bean	Pumpkin
Luffa gourd	Cilantro
Chinese wax gourd	Melon
Round gourd	
Bottle gourd	
Chinese long gourd	
Ridge gourd	
Bitter melon/bitter gourd	
Ash gourd	
Round gourd	
Egyptian spinach	
Malabar spinach	
Sesame	
Fenugreek	
Amaranth	

This study has several limitations to note. First, the small sample size may not fully capture the diversity of experiences and preferences within the Bangladeshi community in Brooklyn. Despite partnering with community organizations and a local community garden for outreach and conducting interviews in-language, the research team was unable to recruit any additional participants willing to be interviewed. However, the research team felt that data saturation was reached and key themes were able to be extracted during analysis, underscoring the relevancy of our findings. Second, the purposeful sample of Bangladeshi participants residing in Brooklyn limit the generalizability of findings to the broader Bangladeshi American population in NYC and the USA, especially as the aim was for the findings to inform development of the Harvest Share Seedlings program. Although our sample represented various gender, age, English proficiency, and immigration experiences, future research should aim to include a larger and more diverse sample to validate and expand upon these findings. Despite these limitations, the study provides valuable insights into the specific needs and preferences of the Bangladeshi community and offers a community-informed approach to develop culturally appropriate food and gardening programs.

## Conclusion

Our study contributes to the limited research among the Bangladeshi community to understand motivators and barriers to participate in food programs. Our formative findings emphasize the promising role of gardening as a method to increase access to fresh culturally appropriate produce, strengthen community bonds, and foster interracial/interethnic understanding for the Bangladeshi community in NYC. The various health, social, and environmental benefits of gardening can make such type of programming appealing to multiple communities, not just the Bangladeshi population, and can also align with priorities of different funding agencies to ensure sustainability of the gardens. Importantly, this community-informed approach represents an inclusive engagement effort that centers community and cultural preferences for health promotion. Future interventions could build on this approach and expand to other racial/ethnic communities. Within the local context of NYC, Harvest Share Seedlings can also be replicated in nearby Brooklyn communities such as Bay Ridge, which has a growing Chinese community that similarly has gardening behaviors and interest in fresh produce.

## Supplementary Material

daag065_Supplementary_Data

## Data Availability

Data available on request from the corresponding author.
