# Supplementary material for: A community-informed approach to develop a gardening model for the Bangladeshi community in Brooklyn, NY
Source: Health Promot Int. 2026 May 25;41(3):daag065. doi: 10.1093/heapro/daag065 (PMC13198943; doi:10.1093/heapro/daag065)
Supplement: daag065_Supplementary_Data [file daag065_supplementary_data.zip › Supplemental File 1 - Interview Guide.pdf]

Interview Guide

General Questions

1. Where do you typically buy your vegetables?
   a. Probes: grocery stores, farmer's market
      i. Can you list stores/locations that you go to? (Store name and cross streets if they don't know exact address)
   b. Is there a primary reason you go there?
      i. Probes: cultural appropriateness, prices, freshness of vegetables, quality of vegetables, clerks speak my language, it's close to my home, it's close to my children's school/work, easy to commute
2. What vegetables do you typically eat at home?
   a. How do you typically prepare them?
      i. Probes: traditional Bangladeshi cooking methods, American cooking methods
   b. How are they served? (e.g., family style, people have own plates)
   c. Who do you typically eat meals with?
3. Are there any vegetables you like but are not able to access?
4. Why are you not able to access these vegetables?
      i. Probes: cost, not available here

Assessing Use of Food Assistance: We understand that access to adequate food is a concern for many individuals and families, especially since the COVID-19 pandemic. We would like to ask you a few questions about your experiences related to food availability and affordability. Your responses will help us better understand the challenges faced by the community and work towards finding solutions to increase access to fresh vegetables. Please feel free to share your thoughts and experiences openly. Your input is valuable, and your responses will be treated with confidentiality and used for research purposes only.

1. Tell me about any experiences related to receiving fruits and vegetables or food assistance. This could include any support you or anyone in your household may have received.
      i. Probe – Food assistance/distribution from sources such as churches/mosques, food banks, food pantries, local community organizations, or government assistance (SNAP/WIC), school meals, senior farmers' market nutrition programs, meals on wheels/meal delivery
   b. If you/your household received food assistance, tell us about your/your household's experience in joining the program.
   c. If you/your household received food assistance, tell us about your/your household's motivation to join the program
      i. Probes: Financial relief, COVID-19 impact, Ease of access
   d. If you/your household have never participated – Why not?
      i. Probes: Misconceptions/stigma, unaware of how to access public/food assistance, unable to access halal options, no organization I trust doing this work, language barriers, do not know that such programs exist. Or not eligible for food assistance program (SNAP)

2. Tell me about your awareness of whether the Bangladeshi community are utilizing food programs.
    a. Probes: Food assistance from sources such as churches/mosques, food banks, food pantries, or government assistance (SNAP/WIC)
    b. [If Bangladeshi community is utilizing food programs] Tell me about your awareness of how the Bangladeshi community is utilizing food programs?
3. Tell me about your familiarity with the SNAP program.
    a. Prompt: Who is eligible to participate in SNAP?
    b. Prompt: What can you use SNAP benefits for?
4. Tell me about your familiarity with the WIC program.
    a. Prompt: Who is eligible to participate in WIC?
    b. Prompt: What can you use WIC benefits for?
5. What factors would make you feel comfortable to participate in a food access program?
        i. Probes: Access to halal accommodations, culturally relevant foods/ fruits and vegetables; Location, receiving information from organizations I trust; dignity, respect in process; discretion for use (e.g., people don't know you're using food assistance programs)

Interest in Food and Gardening Programs: The next set of questions is related to your interest in different types of food-access programming.

1. **Community Garden:** Tell me about your experiences with home gardening or urban gardening activities in NYC
    a. Tell us what motivated you to start gardening
        i. Probes: Lack of cultural vegetables, Cost-effective, used to farm/engage in gardening in Bangladesh, access to fresh vegetables, family value to garden
    a. Are there challenges or obstacles you have faced while engaging in home gardening in NYC?
        i. Probes: Cost, lack of space, local regulation, doesn't have a green thumb, access to seeds
    b. How have you overcome or addressed these challenges?
        ii. If not, then why?
        iii. Probes: Cost, lack of space, local regulation, doesn't have a green thumb
    b. Tell me about your interest in joining a community garden located in Bangladeshi neighborhoods in South Brooklyn/Kensington?
        i. Probes: access to fresh produce, ability to garden, interacting with others who like to garden, being able to grow culturally appropriate vegetables/Bangladeshi vegetables
    c. What location would be most convenient for you to participate in the community garden?
        i. Probes: Community centers, mosques, etc.
        ii. Can you list some examples?
    d. What plants/vegetables do you want to grow in the community garden?
        i. Can you list some examples?

   ii. Would you be interested in growing vegetables that are good for controlling your cholesterol? Some examples include eggplants, okra, Brussel sprouts, cauliflower, broccoli, zucchini, bitter melon, and carrots.
  e. Tell me about your interest in receiving seedlings to grow in your own home.
   i. Would you prefer that over a community garden?
   ii. Can you tell me what seedlings you would be interested in receiving?
2. **CSA**: For the next couple questions, we will be discussing Community Supported Agriculture programs, CSAs for short. In a CSA program, an individual purchases a share from a farm before the growing season for a fixed amount of money and then receives baskets of fresh produce from the farm's harvest at set timepoints throughout the season. This CSA program will provide vegetables that Bangladeshis are familiar with/use in your cooking.
  a. Tell me about your interest in joining a CSA program.
   i. Probes: Access to fresh vegetables, health, produce quality, try new foods, support farmers
  b. What location would be convenient for you?
   i. Probes: Specific neighborhood community center, mosques, etc.
  c. Who in your family would be able to pick up the produce box?
  d. Tell me about how you want to pick up your produce box
   i. Probes: Pick up at farm, pick up from neighborhood community center/mosque, delivery to home
  e. [CSA Payment] How do you plan to pay for the produce box?
   i. Probes: SNAP/WIC, credit/debit card, or cash
  f. Are there any types of vegetables that you would prefer to receive?
   i. Prompt: Can you list some examples?
  g. What types of produce might dissuade you from joining a CSA?
   i. Probes: Non-Bangladeshi or Non-Asian/Unfamiliar vegetables to Bangladeshi
   ii. Prompt: Can you list some examples?
3. **Culturally appropriate farmer's market:** Tell me about your interest in a farmer's market set up in a Bangladeshi neighborhood that sells vegetables you are familiar with/Bangladeshi vegetables?
  a. How often would you access this farmer's market?
   i. Probes: Weekly? Twice a week? Every other week?
  b. What location would be convenient for you?
   i. Probes: Specific neighborhood community center, mosques, etc.
  c. Can you list some vegetables you want to buy at the farmer's market?
  d. What are the payment methods you want to see at this farmer's market?
   i. Probes: Cash, credit/debit card, SNAP
4. Is there any other programming that we didn't discuss today that you would be interested in?
  a. Probes: Community fridges, culturally appropriate food pantries, partnerships with farmers/local farms
5. Can you tell me about any trusted community leaders/organizations/religious institutions whose involvement would encourage you to participate in any of the programs we discussed?
