# Supplementary material for: A community-informed approach to develop a gardening model for the Bangladeshi community in Brooklyn, NY
Source: Health Promot Int. 2026 May 25;41(3):daag065. doi: 10.1093/heapro/daag065 (PMC13198943; doi:10.1093/heapro/daag065)
Supplement: daag065_Supplementary_Data [file daag065_supplementary_data.zip › Supplemental File 2 - Codebook.pdf]

Code	Sub Code	Sub Sub Code	Definition
Vegetable Behaviors	*(no sub code)*		
	Source of veg		Apply this code when participant discusses where they buy their vegetables from
	Reason for veg source		Apply this code when participant discusses why they buy their vegetables at the source they do
	Veg eaten at home		Apply this code when participant discusses vegetables typically eaten at home
	Preparation of veg		Apply this code when participant discusses how they cook their vegetables (i.e. Bangladeshi/American cooking methods)
	Mealtime behavior		Apply this code when participant discusses how vegetables are served (i.e. family style) and who they are eating with
	Inaccessible veg		Apply this code when participant discusses vegetables they are unable to access
	Reason for veg inaccessibility		Apply this code when participant discusses why they are not able to access vegetables
Use of Food Assistance	*(no sub code)*		
	No household usage of food assistance	*(no sub sub code)*	
		Reason for non-usage	Apply this code when participant discusses reasons they do not use food assistance programs (i.e. stigma, no halal options, language barriers, ineligible, etc.)
	Household usage of food assistance	*(no sub sub code)*	
		Source of household food assistance	Apply this code when participant discusses the type of food assistance they received (i.e. food pantries, food banks, SNAP, WIC)
		Satisfaction with food assistance	Apply this code when participant discusses their experience with using a food assistance program -- can include satisfaction, dissatisfaction, stigma, etc.

Code	Sub Code	Sub Sub Code	Definition
		Reason for food assistance	Apply this code when participant discusses reasons they used food assistance programs (i.e. financial, COVID, etc.)
	Community usage of food assistance	*(no sub sub code)*	
		Source of community food assistance	Apply this code when participant discusses where the Bangladeshi community is accessing food assistance programs.
	SNAP familiarity	*(no sub sub code)*	
		Familiar with SNAP	Apply this code when participant discusses that they are familiar with SNAP
		Unfamiliar with SNAP	Apply this code when participant discusses that they do not know about SNAP
	WIC familiarity	*(no sub sub code)*	
		Familiar with WIC	Apply this code when participant discusses that they are familiar with WIC
		Unfamiliar with WIC	Apply this code when participant discusses that they do not know about WIC
	Comfort in participation		Apply this code when participant discusses factors that make them comfortable in participating in food assistance programs (i.e. halal accommodations, partnering with trust organizations, etc.)
Experience with Gardening	*(no sub code)*		
	No prior gardening experience		Apply this code when participant discusses that they do not garden
	Prior gardening experience	*(no sub sub code)*	
		Reasons for gardening	Apply this code when participant discusses reasons that motivated them to garden (i.e. cost, used to garden in Bangladesh, access to fresh vegetables, family/culture values)
		Challenges with gardening	Apply this code when participant discusses challenges they faced gardening in NYC (i.e. space, local regulations, access to seeds etc.)

Code	Sub Code	Sub Sub Code	Definition
		Overcome gardening challenges	Apply this code when participant discusses ways they have overcome any challenges they had towards gardening in NYC
	Knowledge of community gardening		Apply this code when participant discusses how they know/heard/seen the Bangladeshi community involved in home/urban gardening either in NYC or back in Bangladesh
Community Gardens	*(no sub code)*		
	No interest in community gardens		Apply this code when participant discusses that they are not interested in a community garden program for Bangladeshis
	Interest in community gardens	*(no sub sub code)*	
		Access to gardening	Apply this code when participant discusses their interest in community gardens due to gardening opportunities
		Social	Apply this code when participant discusses their interest in community gardens due to social reasons
		Health	Apply this code when participant discusses their interest in community gardens due to health reasons
		Culturally appropriate veg	Apply this code when participant discusses their interest in community gardens due to opportunity to access culturally appropriate vegetables
	Location for community garden		Apply this code when participant discusses locations for a Bangladeshi community garden
	Community garden veg preference		Apply this code when participant discusses plants/vegetables they want to grow in the community garden
	Seedlings	*(no sub sub code)*	
		No interest in seedlings	Apply this code when participant discusses that they are not interested in receiving seedlings
		Interest in seedlings	Apply this code when participant discusses their interest in receiving seedlings and the types of seedlings they want to receive
	Generational Conflict	*(no sub sub code)*	

Code	Sub Code	Sub Sub Code	Definition
		Veg selection	Apply this code when participant discusses generational conflict in choosing what to grow in the community garden
		Decision making authority	Apply this code when participant discusses generational differences in decision-making authority within the gardening program
		Expectations	Apply this code when participant discusses generational conflicts arising from differing expectations and goals for the program
CSAs	*(no sub code)*		
	No interest in CSAs		Apply this code when participant discusses that they are not interested in joining a CSA program for Bangladeshis
	Interest in CSAs	*(no sub sub code)*	
		Community	Apply this code when participant discusses interest in CSAs due to benefits for the community
		Social	Apply this code when participant discusses their interest in CSAs due to social reasons
		Product related	Apply this code when participant discusses interest in CSAs related to produce<br>Include: more variety, freshness, organic, etc.
		Health	Apply this code when participant discusses interest in CSAs due to health reasons
	Location for CSA		Apply this code when participant discusses locations for a Bangladeshi CSA program
	Pick up person		Apply this code when participant discusses who would be the one picking up the produce box
	Method of CSA pick up		Apply this code when participant discusses how they would want to pick up the produce box (i.e. delivery, pick up at farm, pick up at mosque etc.)
	CSA payment		Apply this code when participant discusses how they would pay for the CSA (i.e. cash, SANP/WIC, credit/debit card)

Code	Sub Code	Sub Sub Code	Definition
	CSA veg preference		Apply this code when participant discusses vegetables they want to receive in a CSA produce box
Farmers Market	*(no sub code)*		
	No interest in farmers market		Apply this code when participant discusses that they are not interested in a culturally-appropriate farmer's market for Bangladeshis
	Interest in farmers market		Apply this code when participant discusses that they are interested in a culturally-appropriate farmer's market for Bangladeshis
	Farmers market frequency		Apply this code when participant discusses how often they would go to the farmer's market
	Location for farmers market		Apply this code when participant discusses locations where the farmer's market should be located (i.e. mosques, community centers etc.)
	Farmers market veg preference		Apply this code when participant discusses vegetables that they would buy at the farmer's market
	Farmers market payment		Apply this code when participant discusses the types of payment that they would use at a farmer's market
Other Programming			Apply this code when participant discusses other types of food and gardening programs that they would be interested in (i.e. food pantries, community fridges, etc.)
Potential Partners	*(no sub code)*		
	Community organization		Apply this code when participant discusses that a community organization's involvement would encourage participation
	Religious institution		Apply this code when participant discusses that a religious institution's involvement would encourage participation
